# Associations Between Language, Speech Sound, and Learning Disorders

**DOI:** 10.3390/brainsci16030341

**Published:** 2026-03-21

**Authors:** Chiara Valeria Marinelli, Emiliano Pizzicannella, Marinella De Salvatore, Daniela Sarti, Vincenza Tommasi, Pierluigi Zoccolotti, Luca Andreoli, Elisa Granocchio

**Affiliations:** 1 Cognitive and Affective Neuroscience Lab, University of Foggia, 71122 Foggia, Italy; chiaravaleria.marinelli@unifg.it (C.V.M.); emiliano.pizzicannella@unifg.it (E.P.); vincenza.tommasi@unifg.it (V.T.); 2 Fondazione IRCCS Istituto Neurologico Carlo Besta, 20133 Milan, Italy; daniela.sarti@istituto-besta.it (D.S.); luca.andreoli@iusspavia.it (L.A.); elisa.granocchio@istituto-besta.it (E.G.); 3 Tuscany Rehabilitation Clinic, 52025 Montevarchi, Italy; pierluigi.zoccolotti@crtspa.it; 4 Gianfranco Salvini Foundation ETS, 52025 Montevarchi, Italy; 5 Department of Humanities and Life Sciences, University School for Advanced Studies IUSS, 27100 Pavia, Italy

**Keywords:** dyslexia, dysgraphia, dyscalculia, specific learning disorders, language disorder, speech sound disorder, comorbidity

## Abstract

**Highlights:**

**What are the main findings?**

- Communication and Specific Learning Disorders are often comorbid.
- Speech Sound and Language Disorders show more selective and robust associations with spelling than with reading or math disorders.

**What are the implications of the main findings?**

- Spelling represents a central link between speech, language and learning.
- Clinicians should adopt a multidimensional approach that jointly evaluates deficits within learning and communication domains.

**Abstract:**

**Background and Objectives:** Children with specific learning disorders (SLD) often present a history of speech and language deficits. However, systematic evidence on the co-occurrence among distinct learning and communication disorders remains limited. This study aimed to describe the associations among reading, spelling, and math disorders and their relationships with clinically diagnosed speech sound and language disorders and speech sound disorders in a large, well-characterized clinical sample. **Methods:** 235 3rd- to 8th-grade Italian children with SLD participated in the study. They were categorized in terms of learning (reading, spelling, and math) and comorbid communication disorders (speech sound, and language disorders), according to established diagnostic criteria. Prevalence rates were assessed for each of the resulting subgroups. **Results:** Comorbidity between the three learning disorders was very frequent; 75.4% of children showed different forms of multiple SLDs, with 47.7% presenting a combined reading, spelling, and math disorder. Communication disorders were reported in 40.4% of the sample. Both language and speech sound disorders frequently co-occurred with spelling disorders, whereas associations with isolated reading or math disorders were more infrequent. Additionally, speech sound disorders frequently co-occurred with isolated spelling disorders, whereas language disorders frequently co-occurred with comorbid spelling disorders. **Conclusions:** Consistent with previous evidence, the study shows that learning disorders are highly comorbid with communication disorders. Critically, speech and language disorders are most frequently comorbid with spelling disorder, independent of reading and math deficits, highlighting spelling as a potential key interface between phonology, language, and learning.

## 1. Introduction

The term Specific Learning Disorder (SLD) refers to a series of impairments that affect the acquisition of academic skills, in the absence of poor learning opportunities and cognitive or sensory deficits [1]. SLD can manifest in various ways, affecting reading, spelling, and mathematical competence. These deficits may occur in isolation, but they are often associated with other learning deficits [2,3,4,5,6,7]. For instance, in a large population study, Moll and Landerl [8] demonstrated that combined reading and spelling deficits are as prevalent as their isolated forms. Indeed, recent evidence indicates that the latent dimensions of reading and spelling are very highly correlated (r = 0.96) [9], indicating that association is the rule rather than the exception. Thus, it has also been reported that seemingly “*poor spellers who are good readers*” [10] (p. 108) may perform inefficiently, provided that sensitive measures of reading are used. There is substantial agreement that comorbidity between such complex behavioural disorders is the result of shared etiological and cognitive risk factors. In contrast, the dissociation between these disorders depends on the fact that the underlying cognitive architectures are partially different [11]. For the sake of clarity, in this paper, we will refer to the single manifestations as Reading Disorder (RD), Spelling Disorder (SD), and Math Disorder (MD).

Besides their frequent co-occurrence, SLDs are frequently associated with communication disorders. Although communication disorders and SLDs are different conditions that show different developmental trajectories [12,13,14], several studies have suggested a continuum between early language difficulties and the development of SLDs [15,16,17,18]. In addition, communication disorders are considered a risk factor for reading, spelling [2,14,19,20] and mathematics development [21,22,23]. Indeed, in a recent study, Rinaldi and colleagues [17] found that about a third of school-age children with SLD were diagnosed with a Language Disorder during pre-school years.

### 1.1. Communication Disorders

Communication Disorders include deficits in language, speech, and communication [1]. In this paper, we will refer to two different manifestations: Language Disorder (LD) and Speech-Sound Disorder (SSD).

LD is characterised by persistent difficulties in the acquisition and use of language not attributable to sensory impairments, motor dysfunctions or other neurological conditions. Children with LD are characterised by difficulties in understanding and using words, phrases, and grammatical structures, and are affected in lexical, morphosyntactic, and syntactic comprehension and organisation [24].

SSD is characterised by persistent difficulties with speech sound production, resulting in a limitation in effective communication, with the onset of symptoms during the early developmental period. Children with SSD display either difficulties in articulating phonemes (phonetic deficit) or in the mental representation of speech sounds (phonological deficit) [25]. In about half of cases, SSD is associated with LD [26]. The comorbidity between the two disorders is characterised by both greater severity of productive and receptive language deficits [27,28,29] and a worse outcome for the development of academic learning [30,31,32,33].

### 1.2. Communication Disorders and Reading

The association between communication disorders and SLDs has attracted increasing scholarly attention [2,14,34,35,36,37]. Approximately 25% of children with RD have a documented history of clinically significant speech production difficulties [38,39]. Nevertheless, evidence indicates that expressive phonological deficits are not sufficient for the development of RD [19,28,40,41,42]. For instance, Lewis and colleagues [43] demonstrated that the comorbidity rate between SSD and RD increases from 18% to 75% when LD is also present. Furthermore, an association between RD and LD has been observed independently of SSD, although the prevalence of this association varies considerably. A recent review by Adlof and Hogan [44] reported that the co-occurrence rate of the two disorders ranges from 17% to 71%, depending on the timing of LD diagnosis (pre-school versus school years) and the type of sample (clinical versus unselected). Studies examining the relationship between communication and reading in unselected samples have identified association rates between 15% to 20% [14], whereas studies on clinical samples exhibit higher association rates, approximately 50% [45]. Comparable association rates emerged in studies of children with RD in Italian, a language characterised by a very regular orthography [46,47,48,49,50], which is the object of the present study. Additionally, these studies reported no significant differences in the reading abilities of children with dyslexia with (or without) a prior communication disorder. However, most of these studies relied on retrospective anamnestic reports to characterise language skill profiles (but see [50]).

In summary, the relationships among SSD, LD, and RD are complex and remain incompletely understood. These three conditions frequently co-occur at frequencies higher than expected, likely due to shared genetic and environmental etiological factors [11]. Phonological awareness [51,52,53] and phonological working memory [14,54,55,56] are often identified as key contributors to the association between speech, language and reading disorders. Nevertheless, debate continues regarding the influence of phonology on so-called “pure” disorders [57]. For example, some researchers remain sceptical that dyslexia can be explained solely by phonological impairments [58,59]. It is likely that additional factors contribute to these disorders [43].

### 1.3. Communication Disorders and Spelling

Research examining the relationship between communication and learning has predominantly focused on reading. However, the interrelationship between oral and written language is extensively documented, encompassing both typical [60,61,62,63,64] and atypical developmental trajectories [65,66,67,68,69].

As with reading, phonology is for many a key contributor to the relationship between literacy and language [28,42,67,70,71,72]. Indeed, children diagnosed with SSD face an elevated risk of encountering spelling difficulties [68,73,74]. Moreover, children with isolated SSD exhibit poorer performance in spelling compared to reading [75], probably because spelling requires more complete phonological and orthographic representations than reading does [76].

However, even if there is evidence that phonological deficits are sufficient alone to cause spelling problems [42,77,78,79,80], the results on this issue remain inconclusive [25,75]. In a recent review, Joye and colleagues [67] found that even if phonological impairments alone could lead to spelling difficulties, the severity of these difficulties is exacerbated when SSD coexists with more extensive language impairments. For instance, Lewis and colleagues [43,68] found a higher prevalence of SD when SSD was associated with LD (55%) rather than in “pure” cases of SSD (30%). Data on the prevalence of language problems among children with SD are instead limited. In a study of a Russian-speaking population, a language characterised by a semi-transparent orthography, Rakhlin and colleagues [81] found that 31% of children with SD had been diagnosed with LD at school age.

However, not all research has identified inadequate spelling proficiency among individuals with communication impairments, both in regular [47,48,49,50] and irregular orthographies [13,55,61]. The outcomes may also be influenced by the tasks employed (i.e., spontaneous production vs. dictation) [82] and by the error coding system’s degree of refinement [83]. Indeed, in a recent and targeted study on Italian children with dyslexia and LD, Angelelli and colleagues [46] revealed a worse spelling proficiency when RD was associated with LD, especially with the management of phoneme–grapheme conversion rules in phonologically complex stimuli.

### 1.4. Communication Disorders and Math

The connection between communication and mathematical development is less forthright and relatively less researched, and it is limited to the relationship between MD and LD. Although some basic numerical abilities (such as subitizing) emerge in the early development and have a preverbal origin [84,85], during school-age mathematics learning is carried out through linguistic experiences [86]. Numerical knowledge is, at least in part, organized and constructed through language, and certain language-based processes, such as labelling and scaffolding, are necessary precursors to the development of more complex numerical skills [87,88,89]. Indeed, linguistic and mathematical development have commonalities [22]. For example, efficient phonological processing facilitates the performance of many arithmetic tasks, such as counting [21].

The relationship between communication disorders and mathematics is indeed interesting, given that mathematics involves both verbal and nonverbal representations. In a recent review, Cross and colleagues [21] concluded that communication difficulties create a barrier to mathematical learning in tasks heavily relying on verbal skills (i.e., number transcoding, counting, mental arithmetic, and word-based problems). However, the influence of factors outside of the language domain cannot be ruled out. For instance, the capacity to acquire and consolidate instances (i.e., mnestic traces in declarative memory), which, being implied in math through arithmetical facts retrieval and reading and spelling through lexical representation retrieval, has been called out to explain the comorbidity among SLDs [90,91,92], may also be implicated in lexical/semantic retrieval in oral language.

A frequently underestimated aspect of the association between communication, literacy, and math pertains to the significant rate of comorbidity among SLDs. In a study involving a sample at high risk of developmental difficulties, Snowling and colleagues [23] reported that 67% of children diagnosed with MD also presented an LD. In addition, 65% of the sample exhibited an RD, while one-third manifested all three disorders concurrently.

### 1.5. The Present Study

A comprehensive understanding of the relationships between communication and learning requires an integrated examination of reading, spelling, math, and communication disorders, along with a consideration of their frequent co-occurrence. Addressing this complexity depends upon the availability of a large and clinically well-defined sample, which allows for a detailed and reliable description of the co-occurrence patterns across multiple domains. The present study draws on a large sample of Italian school-aged children with a diagnosis of specific learning disorder (SLD), enabling a fine-grained descriptive characterisation of co-occurrence rates among different learning difficulties. Crucially, participant characterisation is based on formal clinical diagnoses, rather than retrospective anamnestic reports. In particular, communication disorders are identified through an established diagnosis, ensuring a rigorous and methodologically sound classification of participants. This approach enhances the interpretability of the observed association patterns and reduces potential biases related to parental recall or incomplete developmental histories. Notably, the present study adopts a cross-sectional and clinic-based design. Therefore, the analyses are intended to describe patterns of co-occurrence among disorders within a clinically referred sample with SLD, rather than to estimate population prevalence or establish causal or developmental relationships between communication and learning disorders.

Within this methodological perspective, the study follows a three-step descriptive approach. First, we present the patterns of co-occurrence among the three main subtypes of SLD—reading, spelling, and math—irrespective of the presence of communication disorders. Second, we report the prevalence of clinically diagnosed communication disorders within the SLD sample. Finally, we provide a detailed descriptive analysis of the co-occurrence patterns between communication disorders and specific subtypes of SLD, reporting association rates for each subgroup to offer a comprehensive overview of the relationships between communication, reading, spelling, and mathematical difficulties.

## 2. Methods

### 2.1. Participants

The experimental group consisted of a clinical sample of 235 school-age children (134 males, 101 females; mean age = 10.72 ± 1.96; range = 7.88–14.4), attending primary and secondary schools in the Milan metropolitan area. Specifically, the sample involved 65 third graders, 45 fourth graders, 45 fifth graders, 21 sixth graders, 15 seventh graders, and 44 eighth graders.

All children included in the study had a diagnosis of Specific Learning Disorder (SLD). The children were classified according to the type of SLD (i.e., RD, SD, and MD). In addition, they were classified according to the presence of a Communication Disorder (SSD and/or LD). In the following section, we describe the criteria employed for these diagnostic categories.

### 2.2. Diagnostic Classification’s Criteria

The SLD diagnosis was based on the nosographic criteria outlined in the International Classification of Diseases (ICD-10). This approach was appropriately integrated with evidence and recommendations from the Italian National Consensus conference and the most recent Clinical Guideline [93], which introduced standardised clinical thresholds and more refined exclusion criteria. Notably, the operational criteria specified in the Clinical Guideline define separate diagnostic thresholds and procedures for each SLD subtype [93], reflecting the structure of the standardized clinical assessment batteries used in Italian diagnostic practice.

Firstly, for all learning disorders, the following exclusion criteria were adopted: an IQ below the norm (<70), neurological or sensory impairments, psychiatric problems, lack of schooling, and severe socio-cultural disadvantage. Additionally, for each learning disorder subtype, the following criteria were adopted:

Reading Disorder (RD). Reading performance was evaluated using text [94], word, and pseudoword reading tasks [95]. The tasks are described in Appendix A.2. A diagnosis of RD was assigned when a participant performed below the cut-off in least two of the six parameters collected (reading speed and accuracy in word, pseudoword and text reading tasks). The cut-off for reading speed was set at −2 standard deviations, while accuracy was defined as a score below the fifth percentile. Diagnosis was conducted beginning at the end of second grade.

Spelling Disorder (SD). Spelling performance was assessed using two spelling-to-dictation tasks: text dictation and word/pseudoword dictation. The tasks are detailed in Appendix A.3. An SD was diagnosed when a participant scored below the cut-off (fifth percentile) on one of the two tasks administered [96,97]. Diagnoses began at the end of second grade.

Math Disorder (MD). Math performance was assessed through a diagnostic battery [98], which included the following basic math tasks: number transcoding, elaboration of symbolic numerosities, retrieval of arithmetic facts, and mental and written calculation. The tasks are detailed in Appendix A.3. An MD was diagnosed when a participant scored below the cut-off (fith percentile) in at least 50% of the battery tasks. Diagnoses began at the end of third grade.

The diagnosis of SSD and LD was based on the criteria outlined in the international classification manuals available at the time of the assessment [1]. Exclusion criteria included an IQ below the norm (less than 70), as well as the presence of neurological or sensory impairments, psychiatric issues, and severe socio-cultural disadvantages. A pediatric speech-language pathologist assessed speech and language abilities in each child during the preschool years, typically between the ages of 4 and 6. This assessment was part of the clinical pathway leading up to later evaluations for SLDs. Therefore, the LD and SSD diagnoses reported in this study refer primarily to earlier developmental diagnoses documented in the child’s clinical history at the time of SLD assessment. Additionally, all children with an early diagnosis of LD and/or SSD underwent a linguistic reassessment at school age. The reassessment confirmed the initial diagnosis in all cases. We hereby provide our operational criteria for this patient sample as follows:

- Speech Sound Disorder: We conducted a phonological process analysis that included the evaluation of phonetic inventory, phonological processes, and intelligibility estimation [99]. The tasks used are described in Appendix A.5. An SSD was diagnosed when a child’s phonetic inventory fell below average and/or when inappropriate phonological processes were present, such as contrasting, variable, idiosyncratic, unusual processes or a systematic preference for a particular sound.
- Language Disorder: Linguistic performance was assessed using a diagnostic battery [100] that encompassed tasks for sentence and lexical comprehension, sentence and lexical production, as well as narrative/discursive skills. The tasks used are described in Appendix A.6. An LD was diagnosed if a participant scored below average on at least two tasks from the battery. A score below the fifth percentile cut-off was deemed pathological.

### 2.3. Procedures

The testing, carried out for diagnostic and rehabilitation purposes, was administered at Fondazione IRCCS Istituto Neurologico Carlo Besta by paediatric speech-language pathologists. Participants comfortably sat in a quiet room and were tested individually. Testing required 3–4 h. To ensure accurate results and minimise participants’ fatigue, frequent breaks were provided. If necessary, testing was conducted in two days.

### 2.4. Data Analysis

For each disorder studied, participants were categorised based on a dichotomous variable, that indicated whether they had the disorder or not. We calculated the percentage of each disorder and examined the rate of co-occurrence among them. The analyses were primarily descriptive, focusing on characterising patterns of co-occurrence within the clinical sample.

To provide a quantitative estimate of the strength of the associations between disorders, we conducted 2 × 2 analyses. Odds ratios (ORs) with 95% confidence intervals were calculated to evaluate the association between LD and SSD and the presence of specific SLD subtypes (SD, RD, and MD). Chi-square tests were used to assess associations. Fisher’s exact tests were also computed when expected cell counts were small. However, they yielded comparable results. Therefore, χ^2^ statistics are reported for consistency. Lastly, we examined the association between SD status (isolated SD versus comorbid SD) and the diagnostic categories of communication disorders (SLD only, SSD, LD, or SSD + LD) using a χ^2^ test.

## 3. Results

In the following section, we will first describe the co-occurrence patterns between the various SLD subtypes, regardless of communication problems. Then, we will illustrate the prevalence rates of LD and SSD (and their co-occurrence) in the SLD sample. Finally, we will present in more detail how communication problems co-occur with the various subtypes of SLD.

### 3.1. Associations Between SLDs

**Figure 1:**
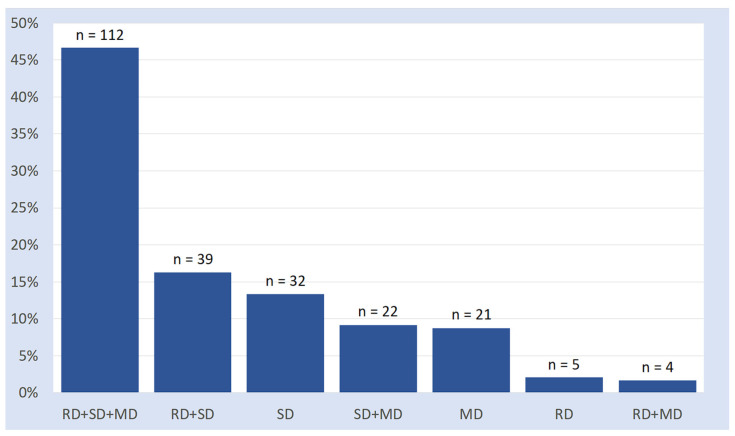
Proportions of SLD’s subtypes (RD: reading disorder; SD: spelling disorder; MD: math disorder).

Data on SLDs are illustrated in Figure 1 and are presented in numerical form in Appendix B (Table A1). Inspection of Figure 1 reveals a series of observations:

- “Pure” cases of SLD (24.6%) are way less frequent than comorbid cases (75.4%).
- Among the “pure” cases, the isolated SD is the most frequent (13.6%), followed by the isolated MD (8.9%) and the isolated RD (2.1%).
- The biggest portion of the comorbid group presents all three learning disorders (reading, spelling, and math, 47.7%).
- The remaining 27.7% of participants are characterised by “partial” comorbidities (two disorders out of three), with the association between reading and spelling disorders being the most frequent (16.6%), followed by that between spelling and math disorders (9.4%). The co-occurrence rate between reading and math disorder is negligible (1.7%).

### 3.2. Prevalence of Communication Disorders in the SLD Sample

**Figure 2:**
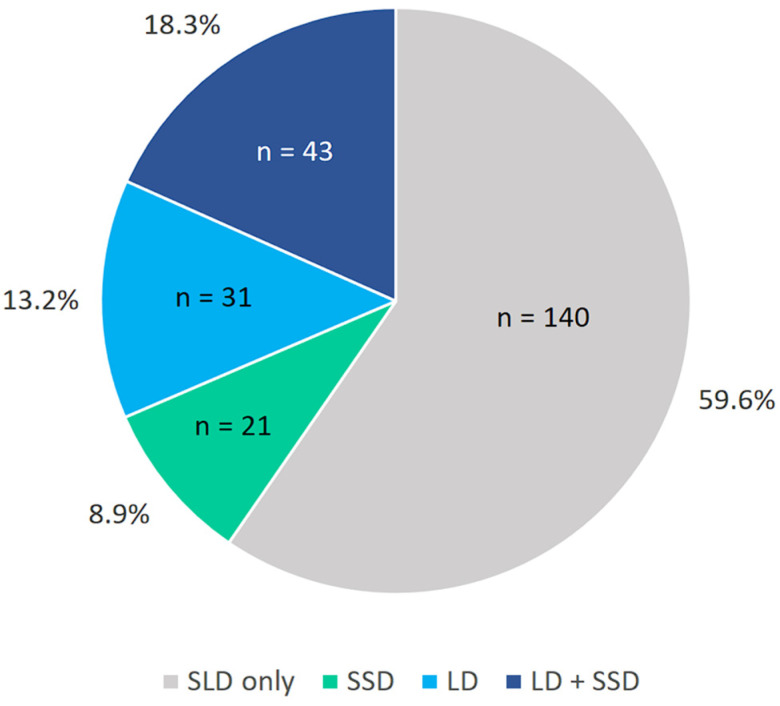
Proportions of communication disorders in the SLD sample (LD: Language Disorder; SSD: Speech Sound Disorder; SLD: Specific Learning Disorder).

Data on communication disorders are illustrated in Figure 2 and are presented in numerical form in Appendix B (Table A2). Inspection of Figure 2 indicates that a considerable portion of children with SLD (40.4%) have a comorbid communication disorder. Specifically, 18.3% had both an LD and an SSD, 13.2% had an SSD without an LD, and 8.9% had an LD without a comorbid SSD.

### 3.3. Associations Between SLD and LD

**Figure 3:**
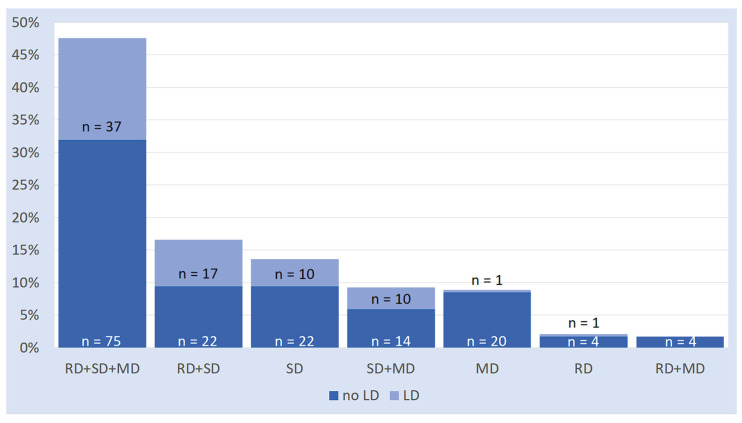
Proportions of LD on SLD subtypes (RD: reading disorder; SD: spelling disorder; MD: math disorder; LD: Language Disorder).

Data on the association between SLDs and LDs are illustrated in Figure 3 and are presented in numerical form in Appendix B (Table A3). Inspection of Figure 3 reveals some key considerations:

- LD co-occurred in 97.4% of cases with a spelling disorder (either “pure” or associated with other SLD subtypes). Only in very few cases (2.6%), it co-occurred with a math or reading disorder without a spelling disorder.
- Conversely, a child with a spelling disorder has a 35.1% chance of having suffered from an LD. Whether or not it is comorbid with other SLDs does not affect this proportion.

To quantify the strength of the associations, we computed the odds ratios comparing the presence of LD in children with and without each SLD subtype (Table 1). Results indicated a significant association between SD and LD: children with SD showed substantially higher odds of presenting LD compared to those without SD (OR = 7.74, 95% CI [1.79, 33.38], χ^2^_(1)_ = 10.10, *p* = 0.001). In contrast, the associations between LD and RD (OR = 1.54, 95% CI [0.83, 2.85], χ^2^(1) = 1.93, *p* = 0.165) and between LD and MD (OR = 0.70, 95% CI [0.39, 1.24], χ^2^_(1)_ = 1.50, *p* = 0.220) were not statistically significant. Overall, these results indicate that SSD co-occurred more frequently than expected by chance with spelling disorder, whereas no reliable associations emerged with reading or math disorders.

### 3.4. Associations Between SLD and SSD

**Figure 4:**
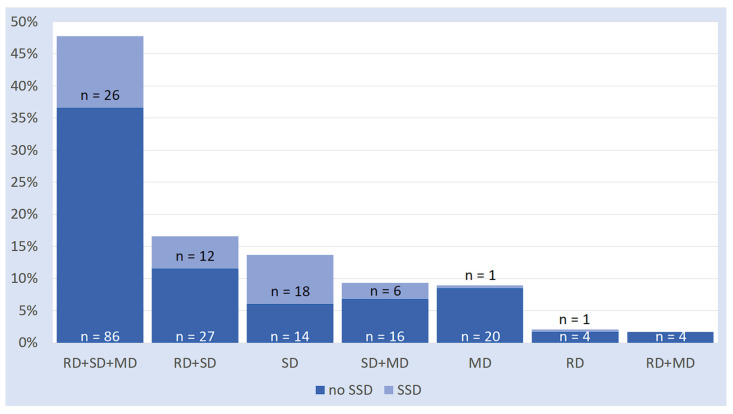
Proportions of SSD on SLD subtypes (RD: reading disorder; SD: spelling disorder; MD: math disorder; SSD: Speech Sound Disorder).

Data on the association among SLDs and SSD are illustrated in Figure 4 and are presented in numerical form in Appendix B (Table A4). Inspection of Figure 4 reveals some key considerations:

- SSD co-occurred in 96.8% of cases with an SD (either “pure” or associated with other SLD subtypes). Only in very few cases (3.2%), it co-occurred with a math or reading disorder without a spelling disorder.
- Conversely, a child with a spelling disorder has a 30.2% chance of having suffered from an SSD. The proportion rises to 56.2% if only the “pure” spelling disorder cases are considered.

To quantify the strength of the associations, we computed the odds ratios comparing the presence of SSD in children with and without each SLD subtype (Table 2). Results indicated a significant association between SD and SSD: children with SD showed higher odds of presenting SSD compared to those without SD (OR = 6.19, 95% CI [1.43, 26.74], χ^2^_(1)_ = 7.54, *p* = 0.006). No significant association emerged between RD and SSD (OR = 0.64, 95% CI [0.35, 1.17], χ^2^_(1)_ = 2.12, *p* = 0.145). In contrast, a significant association was observed for MD (OR = 0.38, 95% CI [0.21, 0.69], χ^2^_(1)_ = 10.56, *p* = 0.001), indicating that children with MD showed lower odds of presenting SSD compared to those without MD. Overall, these results indicate that SSD co-occurred more frequently than expected by chance with spelling disorder, and less frequently with math disorder. No reliable association emerged with reading disorder.

Overall, inspection of Table 1 and Table 2 indicates a strong connection between both LD and SSD with SD. Additionally, Figure 3 and Figure 4 illustrate that LD tends to co-occur more frequently with the spelling disorder when comorbid with other SLDs; in contrast, SSD tends to co-occur most frequently with the isolated form of SD.

**Figure 5:**
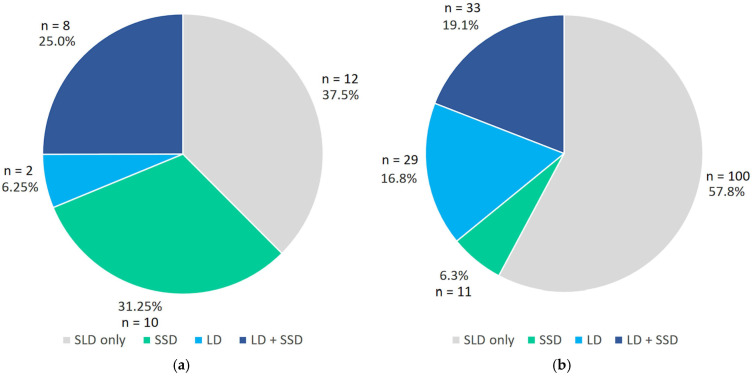
Partial proportions of SSD and LD on Spelling Disorder (SLD: Specific Learning Disorder; SSD: Speech Sound Disorder; LD: Language Disorder). (**a**) Isolated spelling disorder; (**b**) Comorbid spelling disorder.

Figure 5 illustrates this differential pattern. The graph on the left side shows only subjects with a “pure” SD: 46.2% of this group also has SSD (31.2% without LD, 25% with LD), while only 6.2% has LD without SSD. The graph on the right side shows only subjects with a spelling disorder comorbid with other SLDs: 35.9% of this group also have LD (16.8% without SSD, 19.1% with LD), while only 6.3% has SSD without LD. The association between SD status (isolated SD vs. comorbid SD) and the diagnostic category of communication disorders (SLD only, SSD, LD, or SSD + LD) was examined with a χ^2^ test (see Table 3). The analysis revealed a statistically significant association between SD status and diagnostic category (χ^2^_(3)_ = 20.82, *p* < 0.001): children with isolated SD were overrepresented in the SSD category and underrepresented in the LD category relative to expectations. On the contrary, comorbid SD was more prevalent than expected among children with LD, and less prevalent in the SSD group. In the SSD + LD category, the observed frequencies were close to expected values for both isolated and comorbid SDs, indicating that this combined diagnostic group contributed minimally to the overall chi-square statistic. The effect size, measured using Cramér’s V, was 0.32, indicating a moderate association between the variables. Interpretation of these results should be made with caution, as various cells contained expected frequencies below 5, which may inflate the chi-square statistic.

## 4. Discussion

We first examined the rates of association among the SLDs (i.e., reading, spelling, and math disorders), regardless of the presence of communication disorders. As expected, most children (about 75%) had a disorder affecting multiple areas of learning. Indeed, almost half of the sample had all three disorders simultaneously. This result is consistent with the available evidence, which indicates that comorbidity among learning disorders is a prevalent phenomenon [2,5,6,7]. Furthermore, partial comorbidities were asymmetrical, with MD being more frequently comorbid with SD than with RD; this pattern closely resembles the findings by Moll et al. [4] on a large sample of German children.

Isolated SLDs were infrequent but also unevenly distributed. Thus, isolated SDs were considerably more frequent than isolated MDs and even more so than isolated RDs, which comprised only 2.1% of the overall sample.

As for communication disorders, SSD and LD were comorbid in almost 50% of cases, as previously reported [26]. Additionally, children with a communication disorder represented roughly 40% of our SLD sample. Comparing this data with other experimental evidence is challenging for two main reasons. On the one hand, most studies reporting co-occurrence rates between communication and learning disorders have focused on only one type of SLD. This approach could have led to a misrepresentation of the phenomenon, as subjects classified as having an isolated disorder (e.g., reading) may, in fact, be affected in other areas of learning not directly investigated (e.g., spelling and math disorders). Indeed, in the present sample, isolated RD was extremely rare. Another critical issue is that, as noted by Adlof and Hogan [44], the rates of comorbidity reported in the literature are extremely variable, depending on the age and type of sample analysed. In this regard, it is worth remembering that our study involves a clinical sample, which is why the association rates we found may be higher than those found in the general population. We will address this topic in more detail below in Section 4.1. In any case, our results are consistent with those of previous studies on communication disorders and RD, which reported an association rate close to 50% [45].

Overall, our findings on the association rate between communication and learning disorders are broadly consistent with previous literature. However, the results diverge from previous experimental evidence in one potentially relevant aspect. A detailed examination of the learning domain affected (i.e., reading, spelling, or math) revealed that, in this clinical sample, communication disorders most frequently co-occurred with SD. Specifically, children with SD showed substantially higher odds of presenting a communication disorder compared to those without SD. This result was present for both SSD, which co-occurred with SD in 96.8% of cases, and LD, which co-occurred with SD in 97.4% of cases. On the one hand, this finding is consistent with the idea that phonological, lexical, and morphosyntactic skills are essential for spelling competence [9,63,101,102,103]. On the other hand, it seems inconsistent with the idea that RD is due to a phonological core deficit, which would explain their association with communication disorders [14,104]. If this were the case, one would find significant rates of association between communication and reading disorders even in the absence of SD. On the contrary, notable co-occurrence rates between SDs and LDs were present even in the absence of reading deficits.

The significant association found between spelling and communication disorders may be partially attributed to the degree of orthographic transparency in the language under investigation. Several studies in Italian, a language characterised by high orthographic transparency, have reported no significant differences in decoding abilities between children with dyslexia with or without an LD [50] or with or without previous language delays [46,47,48,49]. However, Angelelli and colleagues [46] demonstrated that LD exacerbates spelling deficits when assessed using a sensitive procedure. It is also important to note that Italian orthography is highly transparent in reading but somewhat less transparent in spelling, as several phoneme-to-grapheme correspondences involve alternative spellings and orthographic conventions that introduce a degree of ambiguity. As a result, for Italian-speaking children, accurate spelling is typically more demanding than accurate word reading. This characteristic of the writing system may further contribute to the pattern observed in the present sample, in which spelling difficulties appear particularly associated with communication disorders.

Within the mathematical domain, the relationship between math and communication disorders appears to be complex. Specifically, communication disorders in the sample are comorbid with MDs only when an SD is also present. This observation does not entirely align with existing evidence, which suggests that the association between MD and communication disorders is primarily based on linguistic factors (for a recent review, see [21]). However, this observation should be interpreted with caution. In Italy, children are diagnosed with MD when they show deficits in at least half of the tests within a standardised assessment battery that evaluates various basic mathematical skills. These skills include numerical transcoding, symbolic quantity processing, arithmetic fact retrieval, and both written and mental calculation abilities [93]. Therefore, it remains possible that children with communication disorders who do not have a formal MD diagnosis may still struggle with math tasks that rely more heavily on verbal skills. Moreover, it is important to recognize that individual performance varies along a continuum. The presence of a disorder, along with any comorbidity, also depends on the criteria used for identifying the presence/absence of a deficit [105,106]. As reported in Section 2, the formal criteria for diagnosing different types of SLD in Italy rely on domain-specific diagnostic procedures and thresholds, reflecting the structure of the standardized assessment batteries adopted in clinical practice. As with any diagnostic classification system, the use of specific operational thresholds may influence the observed distribution and co-occurrence of disorders to some extent. Further focused research is needed to clarify this issue more thoroughly.

Finally, given the widespread overlap between communication and spelling disorders, we examined the differences in this association pattern in children with “pure” SD and SD comorbid with other learning deficits. The results indicate that SD manifestation appeared to vary within the sample as a function of co-occurring developmental conditions. Specifically, isolated SD was disproportionately associated with SSD, consistent with other evidence that locates spelling difficulties in phonological representation and phoneme–grapheme mapping deficits when broader language systems are relatively intact [42,77,78,79,80]. In contrast, when SD occurred alongside LD, it was more often comorbid with other SLDs. This pattern suggests an involvement of additional learning and linguistic mechanisms, rather than a circumscribed phonological weakness.

### 4.1. Limitations and Future Directions of Research

Some limitations of the present study should be acknowledged. First, the study adopted a categorical, diagnosis-based approach to characterise communication and learning disorders. This approach allows for a clear and clinically meaningful description of co-occurrence patterns in a large sample. However, it cannot fully capture the dimensional variability of linguistic, reading, spelling, and mathematical skills. Future research should complement diagnostic classifications with direct task-based measures of performance. This approach would allow for a more fine-grained analysis of shared and distinct mechanisms across domains.

Second, the sample included only children with a diagnosis of SLD. In contrast, it did not consider children with communication disorders in the absence of learning disorders. As a result, the present findings provide information on the characterisation of communication disorders within the context of SLD. In contrast, they do not allow for a comparison with profiles of children presenting with isolated communication disorders. Future studies should adopt broader sampling strategies, including children with communication disorders without comorbid learning difficulties, to better disentangle disorder-specific patterns of association.

Third, the sample was drawn from diagnostic/rehabilitation pathways. This form of self-selection may have influenced the observed rates of comorbidities among SLDs as well as different types of SLD and communication disorders. Children with isolated learning difficulties may be less likely to access specialized clinical services if their performance improves with schooling or if their difficulties are somewhat compensated. In contrast, children presenting broader or persistent learning difficulties are more likely to be referred for diagnostic evaluation.

Fourth, the large age range of the participants (3rd to 8th grade) may have introduced a potential developmental bias. In transparent orthographies such as Italian, reading accuracy tends to improve with age due to the high transparency of the orthography. In contrast, spelling difficulties are generally more persistent, particularly in the case of words with ambiguous transcription [107]. Similarly, it is well known that LD tends to persist over developmentally, even though its phenomenology may change over time [108]. Future studies based on larger samples and a larger range of participants’ ages would allow a more detailed examination of whether the observed associations remain stable across different age groups. Analyses stratified by developmental stage could help clarify the extent to which the relationships between communication disorders and specific SLD profiles reflect stable cross-domain links or developmental changes in the clinical manifestation of learning difficulties.

Taken together, these limitations underscore the need for future work integrating diagnostic rigour, dimensional assessment, and broader clinical sampling. At the same time, they highlight the value of the present study in providing a large-scale, diagnosis-based descriptive framework for understanding patterns of co-occurrence between communication and learning disorders.

### 4.2. Conclusions

In conclusion, this study provides a comprehensive description of the co-occurrence patterns between communication and learning disorders in a large, clinically characterised sample of children with SLD. The findings confirm that comorbidity among learning disorders is the rule rather than the exception and that communication disorders are highly prevalent in this population. Beyond this general overlap, a key finding of the study is the strong concentration of communication disorders within spelling disorder profiles in this clinical sample, which emerged independently of reading and math difficulties and extended across both phonological and broader language impairments.

Even though these findings do not allow conclusions to be drawn about the underlying causal mechanisms and etiological pathways, they suggest that spelling may represent an important point of overlap between communication and learning processes, particularly in transparent orthographies, and that communication disorders may exert a more direct impact on spelling than on reading or mathematical performance. The differentiated patterns observed across subtypes of SLD further indicate that the manifestation of spelling difficulties varies as a function of co-occurring developmental conditions, supporting the need for a multidimensional diagnostic perspective.

By leveraging a large sample and relying on formal clinical diagnoses rather than retrospective reports, the present study provides a robust, clinically valid characterisation of the associations between communication and learning disorders. It also underscores the importance of systematically assessing language skills—especially spelling-related processes—in children with learning disorders.

## Figures and Tables

**Table 1:** Association between subtypes of SLD and LD.

SLD Subtype	No-LD	LD	χ^2^_(1)_	*p*	Odds Ratio	95% CI
no-SD	29	2	10.10	0.001	7.74	[1.79, 33.38]
SD	133	71				
no-RD	57	19	1.93	0.165	1.54	[0.83, 2.85]
RD	105	54				
no-MD	49	28	1.50	0.220	0.70	[0.39, 1.24]
MD	113	45				

Note. Values represent observed frequencies. Odds ratios compare the odds of a previous LD diagnosis in children with and without each SLD subtype. SLD: Specific learning disorder; LD: Language disorder; SD: Spelling disorder; RD: Reading disorder; MD: Math disorder.

**Table 2:** Association between subtypes of SLD and SSD.

SLD Subtype	No-SSD	SSD	χ^2^_(1)_	*p*	Odds Ratio	95% CI
no-SD	29	2	7.54	0.006	6.19	[1.43, 26.74]
SD	143	61				
no-RD	51	25	2.12	0.145	0.64	[0.35, 1.17]
RD	121	38				
no-MD	46	31	10.56	0.001	0.38	[0.21, 0.69]
MD	126	32				

Note. Values represent observed frequencies. Odds ratios compare the odds of a previous SSD diagnosis in children with and without each SLD subtype. SLD: Specific learning disorder; SSD: Speech-Sound disorder; SD: Spelling disorder; RD: Reading disorder; MD: Math disorder.

**Table 3:** Association between different forms of SD (isolated or comorbid) and categories of Communication Disorders. Numbers in brackets indicate percentages by column.

	Forms of Communication Disorders				
SD Status	SLD Only	SSD	LD	SSD + LD	Total
Isolated SD	12 (10.7%)	10 (47.6%)	2 (6.5%)	8 (19.5%)	32 (15.6%)
Comorbid SD	100 (89.3%)	11 (52.4%)	29 (93.5%)	33 (80.5%)	173 (84.4%)
Total	112 (100%)	21 (100%)	31 (100%)	41 (100%)	205 (100%)

Note. Values represent observed frequencies, with percentages by column in parentheses. SD = Spelling Disorder; SLD = Specific Learning Disorder; SSD = Speech Sound Disorder; LD = Language Disorder. The chi-square test of independence was significant, χ^2^_(3)_ = 20.82, *p* < 0.001, Cramér’s V = 0.32.

## Data Availability

The data presented in this study are available on request from the corresponding author due to privacy reasons.

## Abbreviations

The following abbreviations are used in this manuscript:

SLD	Specific Learning Disorders
RD	Reading Disorder
SD	Spelling Disorder
MD	Math Disorder
SSD	Speech Sound Disorder
LD	Language Disorder

## Appendix A

### Appendix A.1. IQ Assessment

The evaluation of global cognitive functioning was assessed using the WISC IV multicomponent scale [109,110]. It consists of 10 main subtests, through which a Total Intelligence Quotient (TIQ) is obtained. A TIQ above 70 was used as an inclusion criterion for the study.

### Appendix A.2. Reading Assessment

Text reading task [94]: the subject is asked to read aloud a text appropriate to their education level, within a time limit of 4 min. The parameters considered are reading speed, measured in syllables per second, and the number of errors. Errors may be penalized with one or half a point. One point for incorrect reading of a syllable, omission/addition of a syllable or word, skipping a line, or rereading a line, or pauses longer than 5 s. Self-corrected one-point errors and accent shifts are penalized with half a point. Self-corrections on half-point errors are not penalized. One-point errors that do not change the meaning of the sentence are penalized half a point. Repeated errors on the same word present several times in the text are penalized only once.

Word reading task [95]: The subject is asked to read four lists of words with different levels of imaginability and frequency of use. The syllables per second are calculated for the total of the four lists, as well as the total number of errors. Each mistake is penalized with one point. Self-corrections are not penalized. The syllables per second are calculated on the total of the four lists, as is the number of errors. These parameters are then compared with the normative sample.

Pseudoword reading task [95]: The task consists of three lists of pseudowords of varying lengths, from 5 to 9 graphemes, used to test phonological decoding without relying on visual recognition and semantic mediation. Each mistake is penalized with one point. Self-corrections are not penalized. The syllables per second are calculated on the total of the three lists, as is the number of errors. These parameters are then compared with the normative sample.

### Appendix A.3. Spelling Assessment

Text dictation task [97]: The text dictation consists of a story graded according to the educational level. The total number of errors is calculated. Only one error can be counted for each word. No time constraints are provided.

Word and Pseudoword dictation task [96]: In the dictation of words and pseudowords, the stimuli are 160, divided into 4 sets, and are modulated for phonetic-phonological complexity. Set 1: 70 words with regular transcription, in which the phoneme–grapheme correspondence is 1:1. Set 2: 10 regular words with non−1:1 phoneme–grapheme correspondence, that is, words with syllabic-based conversion. These stimuli require considering the following vowel to determine the correct transcription of a consonantal sound (for example, ‘C’ is written as such when followed by the vowels ‘a’, ‘o’, and ‘u’ as in ‘CASA’ [House], but it is written ‘CH’ when followed by ‘e’ or ‘i’, as in ‘CHILO’ [Kilo]). Set 3: 55 words with ambiguous transcription, which cannot be written based solely on the phoneme–grapheme conversion route, but require the retrieval of the orthographic representation of the word. Set 4: 25 pseudowords with 1:1 phoneme–grapheme correspondence. The accuracy score is calculated for each section and for the whole task. A point is awarded for each correctly spelled stimulus. Self-corrections are not penalized.

### Appendix A.4. Math Assessment

The tasks used are all included in the BDE-2 battery [98].

Counting: The subject is first asked to count forward (from one to forty for 3rd graders, and from eighty to one hundred and forty for 4th to 8th graders). The time taken is recorded. The subject has to count backward within the time spent counting forward. One point is awarded for each number correctly pronounced in this condition.

Number Reading: The subject must read aloud a list of numbers (maximum five digits for 3rd graders, maximum six digits for all other grade levels) in one minute. One point is awarded for each number read correctly.

Number Writing: The subject has to write down a list of numbers in digits (maximum five digits for 3rd graders; maximum six digits for all other grade levels). One point is awarded when it is written correctly.

Multiplication Tables: The subject is asked to perform several multiplication tables in random order. A maximum of 3 s is conceded for each item. One point is awarded for each correct answer.

Direct multiplication Tables: The subject is asked to recite in sequence two specific multiplication tables (4 and 7). A maximum of 3 s is allowed for each. One point is awarded for each correct result given within the time limit. This task is administered only to 3rd graders.

Mental Calculation: The subject is asked to mentally perform a series of additions and subtractions. A maximum of 30 s is conceded for each item. One point is awarded for each correct answer.

Rapid Calculation: The subject has to write the result of a series of simple arithmetic calculations. A maximum of 2 min is conceded to complete the task. One point is awarded for each correct answer. The task is administered only in 4th to 8th grade

Triplets: The subject has to identify which of three numbers is the largest. A maximum of 2 min is conceded to complete the task (3 min for 3rd graders). One point is awarded for each correct answer.

Insertions: The subject has to place a target number on a line containing other numbers, according to its magnitude. A maximum of 2 min is conceded to complete the task. One point is awarded for each correct answer.

Algebraic Sign: The subject has to guess the correct algebraic sign for a given calculation. A maximum of 3 min is conceded to complete the task. One point is awarded for each correct answer. This task is administered only to 3rd graders.

Approximate Calculation: The subjects must estimate the result for a given calculation from four alternatives. A maximum of 2 min is conceded to complete the task, so that the subject is encouraged to rely only on estimation rather than exact calculation. One point is awarded for each correct answer. The task is administered only in 4th to 8th grade.

### Appendix A.5. Speech Assessment

The assessment included an analysis of connected speech through the battery PFLI [99]. The battery consists of a structured picture-based elicitation procedure designed to obtain samples of spontaneous speech, containing all Italian phonemes in different word positions and phonotactic contexts. Children were asked to name or describe visually presented stimuli, and their speech productions were transcribed and analysed to derive phonetic inventories and phonological accuracy measures (e.g., phonological patterns and consonant accuracy), providing a detailed profile of phonological development.

### Appendix A.6. Language Assessment

The tasks used are all included in the BVL 4-12 battery [100].

Lexical comprehension: Lexical comprehension was assessed through a word–picture matching task. Participants were required to select, from an array of pictures, the image corresponding to a verbally presented target word. One point is awarded for each correct answer. This subtest evaluates receptive vocabulary and access to lexical–semantic representations.

Naming (Denomination): Expressive lexical abilities were assessed using a picture naming task. Children were asked to name visually presented objects and actions. One point is awarded for each correct answer. Performance reflects lexical retrieval abilities and word production accuracy.

Sentence Comprehension: Morphosyntactic comprehension was assessed using a sentence–picture matching task. Participants listened to sentences with increasing syntactic complexity and selected the picture that best matched the sentence’s meaning. One point is awarded for each correct answer. This subtest evaluates syntactic parsing and grammatical comprehension.

Sentence repetition: Sentence repetition was assessed by asking participants to repeat auditorily presented sentences of increasing length and morphosyntactic complexity. One point is awarded for each correct answer. This task evaluates the integrity of phonological short-term memory as well as morphosyntactic processing and sentence-level encoding.

Pseudoword Repetition: Phonological working memory and phonological assembly skills were assessed through repetition of pseudowords. One point is awarded for each correct answer. As pseudowords lack semantic representations, this task specifically evaluates phonological processing and short-term memory.

## Appendix B

**Table A1:** Counts and percentages of SLD’s subtypes.

Group	Counts	% of Total
RD	5	2.1%
SD	32	13.6%
MD	21	8.9%
RD + SD	39	16.6%
RD + MD	4	1.7%
SD + MD	22	9.4%
RD + SD + MD	112	47.7%
Total	235	100%

Note. RD = Reading Disorder; SD = Spelling Disorder; MD = Math Disorder.

**Table A2:** Prevalence of LD and SSD in the SLD sample.

LD Status	No SSD	SSD
no LD	140 (81.9%)	21 (32.8%)
LD	31 (18.1%)	43 (67.2%)
Total	171 (100%)	64 (100%)

Note. Values are frequencies with column percentages in parentheses. SLD = Specific Learning Disorder; LD = Language Disorder; SSD = Speech Sound Disorder.

**Table A3:** Distribution of SLD subtypes by Language Disorder (LD) Status.

Group	No LD	LD
RD	4 (2.5%)	1 (1.3%)
SD	22 (13.7%)	10 (13.5%)
MD	20 (12.4%)	1 (1.3%)
RD + SD	22 (13.7%)	17 (23.0%)
RD + MD	4 (2.5%)	0 (0.0%)
SD + MD	14 (8.7%)	8 (10.9%)
RD + SD + MD	75 (46.5%)	37 (50.0%)
Total	161 (100%)	74 (100%)

Note. Values are frequencies with column percentages in parentheses. SLD = Specific Learning Disorder; RD = Reading Disorder; SD = Spelling Disorder; MD = Math Disorder; LD = Language Disorder.

**Table A4:** Distribution of SLD subtypes by Speech Sound Disorder (SSD) Status.

Group	No SSD	SSD
RD	4 (2.3%)	1 (1.6%)
SD	14 (8.2%)	18 (28.1%)
MD	20 (11.7%)	1 (1.6%)
RD + SD	27 (15.8%)	12 (18.8%)
RD + MD	4 (2.3%)	0 (0.0%)
SD + MD	16 (9.4%)	6 (9.4%)
RD + SD + MD	86 (50.3%)	26 (40.6%)
Total	171 (100%)	64 (100%)

Note. Values are frequencies with column percentages in parentheses. SLD = Specific Learning Disorder; RD = Reading Disorder; SD = Spelling Disorder; MD = Math Disorder; SSD = Speech Sound Disorder.
